# Effects of Ecological Dynamics Approach in Physical Education on Physical Fitness and Types of Physical Activity in Middle School Students: An Exploratory Study

**DOI:** 10.3390/jfmk11020165

**Published:** 2026-04-22

**Authors:** Italo Sannicandro, Luigi Armiento, Nicola Trotta, Federico Abate Daga

**Affiliations:** 1 Department of Clinical and Experimental Medicine, University of Foggia, 71122 Foggia, Italy; 2 Department of Neurosciences, Biomedicine and Movement Sciences, University of Verona, 37129 Verona, Italy; 3 Department of Clinical and Biological Sciences, University of Turin, 10124 Turin, Italy

**Keywords:** physical education, small-sided games, ecological dynamics approach, motor skills, physical fitness, perceived physical fitness, student health, schools

## Abstract

**Background**: This study aimed to examine whether a physical education program based on the ecological dynamics approach, implemented through small-sided games (SSG), produces greater improvements in motor skills, daily physical activity levels, and perceived physical fitness in middle school students. **Methods**: Forty-eight students were assigned to an SSG group (ecological dynamics lessons including small-sided games, *n* = 26) or a Control group (traditional lessons based on teacher-centered instruction and analytical exercises, *n* = 22). The intervention lasted 12 weeks, with two sessions per week. Motor performance was assessed using the standing broad jump, 5-standing broad jump, 20 m sprint, 10 × 5 m shuttle run, 5-0-5 agility test, and sit-and-reach test. Daily physical activity was evaluated using the International Physical Activity Questionnaire—Short Form (IPAQ-SF), and perceived physical fitness was assessed using the Visual Analogue Fitness Perception Scale for Adolescents (FPVASA). **Results**: Significant group-by-time interactions were found in all motor tests. IPAQ-SF data revealed significant group-by-time interactions for vigorous and moderate physical activity. Perceived physical fitness showed significant group-by-time interactions for all items except flexibility. **Conclusions**: Physical education lessons structured according to the ecological dynamics approach and implemented through SSG-based protocols led to greater improvements than traditional methods. The dynamic and variable nature of SSG likely enhances neuromuscular stimulation, motor engagement, and motivation during physical education lessons.

## 1. Introduction

In November 2020, the World Health Organization (WHO) presented new guidelines for promoting physical activity and reducing sedentary lifestyles, updating the previous 2010 recommendations with the aim of providing clear and scientifically based guidance on healthy movement behaviours. The WHO guidelines recommend at least 60 min of moderate-to-vigorous physical activity (MVPA) per day for children and adolescents [1]. However, the literature highlights moderate activity levels among young people, with an increasing number not meeting these recommendations [2,3]. Sedentary behavior is recognized as a major public health risk [4], and chronic non-communicable diseases (NCDs) have become the leading global cause of death [4,5]. Regular physical activity significantly reduces the risk of cardiovascular disease, hypertension, diabetes, and several cancers [5,6]. In this scenario, monitoring physical activity and related perceptions is essential to better understand adolescents’ movement behaviors and to evaluate the effectiveness of educational interventions. Questionnaires such as the International Physical Activity Questionnaire (IPAQ) and assessment tools like the Fitness Perception Scale for Adolescents (FPVSA) are widely used and considered reliable for monitoring physical activity and perceived physical fitness [6,7,8,9]. These tools are inexpensive, easy to administer, and commonly adopted in physical education settings [8,9]. Increasing physical activity levels requires interventions in both leisure-time and institutional educational environments. Consequently, the focus is shifted from promoting physical activity alone to designing learning contexts that facilitate daily movement [3]. Schools play a crucial role in promoting healthy motor habits and preventing NCD-related risk factors from early childhood [1]. The identification of curricular activities and the quality of teaching interventions are supported by teacher training programs and active teaching methodologies [10,11]. 

Modern pedagogical approaches increasingly emphasize student-oriented learning and active methodologies. This shift generated a debate within the educational field [12,13] and specifically regarding teaching styles in school-based physical education [14]. Teaching style refers to the decision-making process shared between teacher and student regarding how, when, and why pedagogical choices are made [15,16]. The teaching-learning process is based on the study of teacher-student and student-environment relationships [17]. Among the functional skills required for physical education teachers, the manipulation of teaching styles and the selection of motor tasks are essential [18]. Variations in communication and instructional style influence children’s learning processes [16]. In this context, physical education curricula must respond to the decline in motor skills and promote higher levels of physical activity among adolescents, given the documented reductions in basic motor skills, coordination, and cardiorespiratory fitness [19]. Increasing physical education time, improving teaching quality, and adopting active methodologies are key strategies to counteract this decline [20].

In this perspective, the ecological dynamics approach is recognized as an effective pedagogical framework in physical education [11,21,22,23]. The ecological dynamics approach integrates principles from ecological psychology and dynamical systems theory, emphasizing the continuous interaction between the individual, the task, and the environment [21,23]. Within this framework, learning emerges through the exploration of affordances, where perception and action are tightly coupled.

In physical education, this approach promotes adaptability, variability, and decision-making by designing representative learning environments rather than focusing on the repetition of predefined technical skills. Students are continuously required to adapt their motor behaviour in response to changing constraints, increasing engagement and active participation during lessons [11,21,22,23].

This pedagogical approach is consistent with student-centred teaching styles and lesson structures that increase active learning time. Adolescents are continuously required to adapt their motor behaviour during activities, thereby reducing passive time and increasing engagement [23,24]. Adapting motor behaviour is a key element of this methodology [25], and previous literature has shown that the ecological dynamics approach may lead to greater physical efficiency than directive and prescriptive methods [23,24].

Importantly, this approach is grounded in the specific characteristics of the learning context, taking into account students’ age, skill level, and the constraints of the school environment, thus ensuring its applicability within real educational settings.

In contrast, traditional physical education approaches are typically characterized by teacher-centred instruction, the decomposition of skills into isolated technical components, and the repetition of standardized movement patterns. In these approaches, learning is often structured around the reproduction of ideal techniques in relatively stable and controlled environments, with limited emphasis on variability and decision-making processes. Within the context of the present study, the traditional approach refers to this type of structured and prescriptive teaching, which contrasts with the ecological dynamics approach based on adaptability, interaction with constraints, and problem-solving in dynamic contexts [11,21,23].

In school settings, this approach is typically implemented through structured exercises, analytical drills, and teacher-led activities, with limited emphasis on variability, decision-making, and student autonomy.

To apply the ecological dynamics approach in youth educational contexts, small-sided games (SSG) represent an effective pedagogical tool. SSG is based on a smaller number of participants, a reduced field size, and modified rules while maintaining the essential features of the sport. They are particularly suited to school contexts because they require continuous adaptations to environmental variability and active problem-solving [26,27]. The literature has underscored their effectiveness in improving physical efficiency and in optimizing physiological responses in shorter exercise times [26]. A recent systematic review and meta-analysis highlighted the benefits of SSG on cardiorespiratory fitness, body composition, and metabolic parameters, including lipid profile and insulin sensitivity [27]. These benefits are especially relevant in childhood, as SSG stimulates fitness development in enjoyable contexts and contributes to the prevention of chronic diseases associated with sedentary behavior [26,27,28].

In light of this, several school-based interventions incorporated SSG during physical education classes. For example, FIFA 11 for Health [29] is an interesting protocol that integrates SSG-based interventions with health education. Previous studies have demonstrated its effectiveness in improving aerobic capacity, motor skills, health knowledge, and psychological well-being [4,30,31].

Physical education may therefore influence adolescents’ health and daily habits inside and outside school. This consideration is particularly relevant in Italy, where many adolescents exhibit high sedentary levels [3], and the school is often the only structured opportunity for daily physical activity. Moreover, many students encounter a specialist physical education teacher for the first time during middle school, benefiting from pedagogical and didactic skills that support tailored interventions [32].

Despite growing interest in the ecological dynamics approach, limited evidence exists regarding its effects in school-based physical education, particularly in adolescent populations. In addition, previous studies have mainly focused on performance or physiological outcomes in sport-specific contexts. At the same time, their impact on students’ daily physical activity behaviors and perceived physical fitness remains underexplored. Furthermore, few studies have directly compared ecological-dynamics-based interventions with traditional teaching approaches in real school settings. This gap highlights the need to better understand how different pedagogical approaches influence both motor development and lifestyle-related behaviors in adolescents.

In this study, motor skills, daily physical activity levels, and perceived physical fitness were selected as key outcome variables because they represent complementary dimensions of physical education effectiveness and align with the principles of the ecological dynamics approach.

Therefore, the aim of this study was to examine whether a physical education program based on the ecological dynamics approach, implemented through small-sided games, leads to greater improvements in motor skills, daily physical activity levels, and perceived physical fitness compared to traditional physical education lessons. This study contributes to the literature by providing evidence from a real school-based context and by comparing two distinct pedagogical approaches in adolescents.

We hypothesized that students exposed to the ecological dynamics approach would show greater improvements in motor skills, daily physical activity levels, and perceived physical fitness compared to those following a traditional instructional approach.

## 2. Materials and Methods

### 2.1. Study Participants

The total sample included 48 middle school students (age: 13.41 ± 0.50 years; weight: 63.18 ± 0.79 kg; height: 163.10 ± 0.52 cm; BMI: 23.86 ± 1.88 kg/m^2^). The sample included 23 males and 25 females.

In addition, sample size was estimated using G*Power (version 3.1.9.7, Düsseldorf, Germany). The required sample size was 41 participants (effect size f = 0.35, α = 0.05, power = 0.90) for a repeated-measures ANOVA (group × time). The selected parameters were based on the expectation of a medium-to-large effect size and on commonly accepted methodological criteria to reduce the risk of Type I and Type II errors.

Forty-eight participants were recruited as several classes were involved in order to reflect the real context and prevent selection bias.

A convenience sampling approach was adopted, involving intact classes from the same public middle school. No random selection was applied in order to preserve the ecological validity of the educational setting. As all participants were recruited from the same school, they shared a similar sociocultural and educational context. This homogeneity may have reduced potential variability related to environmental and social factors influencing physical activity behaviours.

The students were assigned to the SSG group (SSG) (*n* = 26; age: 13.42 ± 0.51 years; weight: 62.62 ± 3.60 kg; height: 163.47 ± 2.61 cm; BMI: 23.46 ± 1.63 kg/m^2^) or the control group (CON) (*n* = 22; age: 13.33 ± 0.49 years; weight: 63.73 ± 3.57 kg; height: 162.73 ± 3.45 cm; BMI: 24.11 ± 1.88 kg/m^2^). Group allocation was performed at the class level to preserve the ecological validity of the school setting. Baseline comparisons between groups revealed no significant differences in age, height, weight, or BMI (*p* > 0.05).

Inclusion criteria were: (i) attendance of at least 80% of lessons; (ii) absence of injuries during the intervention period; and (iii) participation in all assessment sessions. Thirteen participants were excluded due to not meeting these criteria.

Written informed consent was obtained from the students’ parents or legal guardians prior to participation, and the study was conducted in accordance with the Declaration of Helsinki and approved by the Ethics Committee of the University of Foggia (protocol code 0349SM/2025, approved on 23 October 2025).

### 2.2. Study Organization

The study followed a quasi-experimental design with two groups (SSG vs. CON) and two time points (pre- and post-intervention). This design was selected to reflect real school conditions and to allow comparison between different teaching approaches over time.

The intervention lasted 12 weeks, with two physical education sessions per week. Each session followed the standard school schedule (approximately 60 min per lesson). The duration and frequency of the intervention were selected in accordance with previous school-based physical education studies, which have shown that programs lasting 8–12 weeks with regular weekly sessions are effective in improving motor skills and physical fitness in adolescents.

The SSG group participated in physical education lessons based on the ecological dynamics approach. Approximately 50% of each session (approximately 20–25 min) was dedicated to small-sided games (e.g., handball, football, basketball, 2 vs. 2, 3 vs. 3, 4 vs. 4 formats with scoring objectives; tasks included maintaining ball possession under numerical superiority/inferiority, scoring under time constraints, and rule-modified games to manipulate tactical complexity). These activities were designed to promote decision-making, adaptability, and continuous engagement through variable and representative learning environments. The remaining part of the lesson included warm-up, cooldown, and complementary activities consistent with the pedagogical approach (e.g., task variability, problem-solving tasks, and guided discovery). Teachers provided guided feedback during the sessions to facilitate learning, while students were encouraged to actively engage in problem-solving situations.

The control group followed a traditional physical education approach characterized by teacher-centered instruction, including analytical exercises focused on technical skill execution (e.g., sport-specific drills such as passing, shooting, and coordination tasks), as well as individual and pair-based activities aimed at improving general physical fitness. The lesson was divided into main phases: warm-up, central phase, cooldown, and stretching. The main instructional characteristics of the two approaches are summarized in Table 1.

Lessons in both groups were delivered by qualified physical education teachers with similar teaching experience (approximately 15 years). The intervention was designed collaboratively by the research team and the teachers to ensure consistency and ecological validity.

Motor and questionnaire assessments were conducted during regular school hours in the first two lessons before and after the intervention. The order of tests was randomized to minimize fatigue and learning effects.

### 2.3. Measures

The participants’ motor skills were assessed in the first two lessons prior to and in the two lessons following the physical education program, which was delivered using either an ecological dynamics approach or a traditional approach.

#### 2.3.1. Physical Fitness Tests

The selected assessment tools were chosen to evaluate the main outcomes of the study, namely motor skills, daily physical activity levels, and perceived physical fitness. Physical fitness tests were used to assess motor skills. These tests are widely used in youth populations. They are commonly adopted in school-based physical education research, as they provide practical and reliable measures of motor performance components such as strength, speed, agility, and flexibility.

##### Standing Broad Jump (SBJ)

The standing broad jump was used to assess lower-limb explosive strength. The subject, positioned behind a starting line, performed a maximum forward jump. The distance was measured from the front edge of the starting line to the rearmost point of contact (usually the heel) reached on landing. Three attempts were allowed, and the best performance, expressed in centimeters, was recorded (ICC = 0.87) [33].

##### 5 Standing Broad Jumps (5SBJ)

The 5 standing broad jump was used to estimate the ability to express repeated power in the lower limbs; a protocol based on five consecutive standing long jumps was administered. The participant performed the jumps in sequence, starting from a static position and landing with both feet, trying to cover the maximum total distance. The total distance covered after the fifth jump was measured, with a recovery time of 60 s between trials. Three attempts were allowed, and the best performance, expressed in centimeters, was recorded (ICC = 0.81) [33].

##### 20 m Sprint Test

A 20 m sprint test was used to assess linear speed. The subject, from a standing position with one foot behind the starting line, performed a maximal-effort sprint to the finish line as quickly as possible. The trial time was measured using photocells (Globus Treviso, Codognè, Italy). Three trials were performed, separated by a complete recovery of approximately 3 min, and the best time in seconds was recorded (ICC = 0.86) [34].

##### Eurofit Shuttle Run Test 10 × 5 m

The 10 × 5 m Eurofit shuttle run test was used to estimate speed and agility. The subject ran back and forth between two lines 5 m apart, making 10 changes of direction (5 out and 5 back), touching the line with their foot at each. The total time, expressed in seconds, was recorded using photocells (Globus Treviso, Italy). Three trials were performed, separated by a complete recovery of approximately 3 min, and the best time in seconds was recorded (ICC = 0.82) [34].

##### 5-0-5 COD Test

The 5-0-5 COD test was used to assess change-of-direction speed. Participants from point A sprint for 15 m to point C, where they make a 180° turn and sprint up to point B. According to the plan, four repetitions were performed, two turns with the right foot, and two turns with the left foot. In any case, the best time was used. A photocell (Globus Treviso, Italy) at point B (line) was used to measure time. Three trials were performed, separated by a complete recovery of approximately 3 min, and the best time in seconds was recorded (ICC = 0.84) [35].

##### Sit-and-Reach Test

The sit-and-reach test was used to assess the flexibility of the posterior chain, performed with a special graduated box. The subject, sitting on the ground with legs extended and feet resting against the graduated box, performed a slow, controlled forward bend, pushing their hands as far as possible along the graduated scale, maintaining the position for about 2 s. Three attempts were allowed, recording the best distance achieved in centimeters (ICC = 0.90) [36].

#### 2.3.2. Questionnaires

In addition, questionnaires were used to assess daily physical activity levels and perceived physical fitness. Specifically, the IPAQ-SF was used to evaluate physical activity levels, while the FP VAS A scale was used to measure perceived physical fitness.

##### IPAQ-Short Form

The International Physical Activity Questionnaire—Short Form (IPAQ-SF) was administered to assess the participants’ levels of physical activity. This questionnaire discriminates between the type and amount of physical activity that the individual normally performs. The questions, relating to activity carried out in the last 7 days, are designed to identify all types of activity performed by the individual. The short version records activity with four levels of intensity: 1. Vigorous activity, 2. Moderate activity, 3. Walking, and 4. Sitting [37].

##### Visual Analogue Fitness Perception Scale for Adolescents

The Visual Analogue Fitness Perception Scale for Adolescents (FPVASA) was used to assess subjective fitness perception [9], as it is inexpensive and quick to complete [8,9]. Specifically, the aim of FPVASA is to analyse how each subject perceived their overall fitness level and its main components by measuring four items: general fitness, cardiorespiratory fitness, muscular strength, speed-agility, and flexibility [9].

### 2.4. Statistical Analysis

Means and standard deviations were reported as descriptive statistics for all analyzed variables. The normality of the data distribution was assessed using the Shapiro-Wilk test, and Levene’s test was conducted to verify homogeneity of variances. A two-way mixed-design ANOVA with repeated measures ([SSG vs. CON] × [pre vs. post]) was conducted to examine effects on physical fitness and questionnaire outcomes. When a significant main effect or interaction was detected, pairwise comparisons were conducted using post hoc tests with Bonferroni correction. The effect size was calculated and interpreted as follows: trivial: 0.00–0.19; small: 0.20–0.59; moderate: 0.60–1.19; large: 1.20–1.99; very large: ≥ 2.00 [38]. All statistical analyses were performed with JASP software (version 0.19.2, Amsterdam, The Netherlands). The selected statistical approach was considered appropriate for the quasi-experimental design and sample size, allowing the analysis of interaction effects while maintaining interpretability without overfitting the data.

## 3. Results

### 3.1. Motor Skills

Overall, significant time × group interactions were observed across all motor skill variables, indicating greater improvements in the SSG group than in the control group.

A significant time × group interaction was found for the standing broad jump (F = 78.240, *p* < 0.001), with a very large improvement in the SSG group (ES = 4.06) and a moderate improvement in the control group (ES = 0.86).

Similarly, a significant interaction was observed for the 5 standing broad jump (F = 80.490, *p* < 0.001), with a very large increase in the SSG group (ES = 3.85), whereas no significant change was observed in the control group (ES = 0.34).

For the 20 m sprint test, a significant interaction effect was found (F = 23.863, *p* < 0.001), indicating a greater reduction in sprint time in the SSG group (ES = 2.28) compared to the control group (ES = 0.84).

A significant interaction was also observed for the 10 × 5 m shuttle run test (F = 58.509, *p* < 0.001), with a very large improvement in the SSG group (ES = 2.21) and a small improvement in the control group (ES = 0.48).

For the 5-0-5 change-of-direction test, a significant interaction was found (F = 6.838, *p* = 0.013), with a large improvement in the SSG group (ES = 1.61), whereas no significant change was observed in the control group (ES = 0.58).

Finally, a significant interaction was observed for the sit-and-reach test (F = 25.788, *p* < 0.001), with a large improvement in the SSG group (ES = 1.42) and no significant change in the control group (ES = 0.24).

Detailed results are reported in Table 2.

### 3.2. Daily Physical Activity Levels (IPAQ-SF)

Significant improvements in daily physical activity levels were observed in the SSG group compared to the control group, particularly for vigorous and moderate activities.

A significant interaction was found for vigorous activity (F = 24.085, *p* < 0.001), with a very large increase in the SSG group (ES = 2.19). In contrast, no significant change was observed in the control group (ES = 0.38).

Similarly, a significant interaction was observed for moderate activity (F = 31.384, *p* < 0.001), with a very large increase in the SSG group (ES = 2.19), while no significant change was observed in the control group (ES = 0.24).

No significant interaction effect was found for walking (F = 1.476, *p* = 0.233). A small but significant increase was observed in the control group (ES = 0.20), whereas no significant change was observed in the SSG group (ES = 0.08).

No significant interaction was found for sitting time (F = 3.535, *p* = 0.069). However, a moderate reduction was observed in the SSG group (ES = 0.66), whereas no significant change was observed in the control group (ES = 0.33).

Detailed results are presented in Table 3.

### 3.3. Visual Analogue Fitness Perception Scale for Adolescents (FPVASA)

Perceived physical fitness significantly improved in the SSG group compared to the control group across most variables.

A significant interaction was found for general fitness (F = 23.643, *p* < 0.001), with a large improvement in the SSG group (ES = 1.85) and no significant change in the control group (ES = 0.11).

Similarly, a significant interaction was observed for cardiorespiratory fitness (F = 10.085, *p* < 0.001), with a large improvement in the SSG group (ES = 1.57). In contrast, no significant change was observed in the control group (ES = 0.35).

For muscular strength, a significant interaction was found (F = 54.175, *p* < 0.001), with a very large improvement in the SSG group (ES = 2.51) and no significant change in the control group (ES = 0.37).

A significant interaction was also observed for speed-agility (F = 36.341, *p* < 0.001), with a large improvement in the SSG group (ES = 1.68) and no significant change in the control group (ES = 0.09).

No significant interaction was found for flexibility (F = 3.431, *p* = 0.073). However, a moderate improvement was observed in the SSG group (ES = 0.79), whereas no significant change was observed in the control group (ES = 0.36).

Detailed results are presented in Table 4.

## 4. Discussion

The aim of this study was to assess whether curricular physical education lessons based on the ecological dynamics approach lead to improvements in motor skills, daily physical activity, and perceived physical fitness compared with traditional programs. This is the first study, to our knowledge, to investigate the effects of an SSG-based intervention in Italian middle schools while varying the teaching methodology and the motor tasks proposed during curricular physical education.

As hypothesized, the results highlighted the effectiveness of SSG-based interventions among Italian middle school students. These findings can be interpreted considering the pedagogical characteristics of the ecological dynamics approach, which emphasizes adaptability, interaction with constraints, and active engagement. Such features may explain the greater improvements observed in the SSG group compared to traditional teaching methods.

The improvements observed in explosive strength tests (SBJ and 5 SBJ) indicate that the intermittent, dynamic, and variable nature of SSG-based interventions determines greater neuromuscular engagement than traditional methods, due to the continuous involvement of jumping, landing, and changes of direction [25]. These findings are consistent with previous studies reporting significant improvements in similar parameters among young athletes who have been exposed to the ecological dynamics approach [24,28].

Similarly, the improvements in speed and agility (20 m, 10 × 5 m, 5-0-5 COD test) in the SSG group can be attributed to the nature of the exercises, which require frequent accelerations, decelerations, and high-intensity running to resolve complex and variable motor situations [26]. In line with these findings, previous studies have shown that the ecological dynamics approach enhances movement quality, executive efficiency [23], and change-of-direction abilities by providing constant variability in motor constraints [24,26]. Furthermore, the continuous interaction between teacher and student during the exercises [24] represents a relevant advantage, supporting higher exercise intensity during physical education lessons.

Although no specific flexibility exercises were included, the SSG group showed improvements in the Sit and Reach test compared with the CON. This may be attributed to the variability of execution and the opportunity to perform movements with a wide range of motion during SSG activities. The literature has indicated varying motor solutions may favor improved functional mobility and movement efficiency [25,39].

The results related to extracurricular physical activity are consistent with previous studies that used similar SSG-based interventions and reported increases in physical activity levels [5,30,31]. In addition, programs differing from traditional school activities, such as FIFA 11 for Health [4,29,30,31,40,41,42], have shown positive effects on physiological and fitness-related parameters, including blood pressure, body composition, postural balance, and muscle strength [4,42]. These outcomes are likely associated with increased overall physical activity engagement and higher exercise intensity, supporting the interpretation that SSG-based approaches can promote both active behaviors and broader health-related adaptations.

Similarly, the IPAQ-SF findings confirmed the positive impact of the SSG-based approach on daily physical activity levels, suggesting a possible transfer of school-based benefits into everyday life. This is in line with previous studies showing a positive influence on motor habits and lifestyle behaviors in adolescents [43]. Moreover, game-based approaches also appear effective for motivating students to increase physical activity, as they integrate tactical aspects and motor skills in an enjoyable, problem-solving context [39]. The perceptions of teachers and students confirm that higher enjoyment and autonomy during SSG can effectively affect activity levels [39].

Programs based on recreational and sports activities may also lead to spontaneous increases in physical activity, both during school hours and in unstructured contexts [42,43]. Previous studies have emphasized an active involvement, motivation, and enjoyment elicited by SSG, encouraging spontaneous participation even outside structured lessons [26]. In light of this, the increase in motor engagement in the SSG group may contrast with the sedentary behavior of adolescence.

The findings related to the FP VAS A scale showed improved subjective perceptions of physical fitness in favor of SSG. Students reported feeling fitter regarding general fitness, cardiorespiratory fitness, muscular strength, and speed-agility. These results align with studies showing that significant exposure to dynamic motor activities enhances perceived competence, fitness, and physical well-being in adolescents [8,9]. Further studies support the idea that SSG-based interventions can improve both physical condition and perceived motor competence [28].

To date, no studies have evaluated changes in physical activity using IPAQ-SF and FPVASA in SSG contexts, highlighting the relevance of these findings.

Although the present study provides interesting insights into curricular physical education, some limitations should be considered. No internal load assessment tools, such as heart rate monitors or RPE measures, were used, which could have provided more accurate information on exercise intensity. Similarly, no body composition assessment was conducted, which could have confirmed the effectiveness of the intervention for weight control.

Another limitation is that biological maturation was not assessed. Considering the age of the participants, differences in maturation status may have influenced physical performance outcomes, potentially introducing bias in the interpretation of the results. Furthermore, although the ecological dynamics approach primarily emphasizes the development of motor competence, adaptability, and decision-making processes, the present study focused on the assessment of physical performance outcomes. This may represent a limitation, as performance-based measures may not fully capture the complexity of competence development within this framework.

Another limitation concerns comparisons between groups exposed to different types of activities and to different teaching approaches. Although the aim of the study was to compare pedagogical methodologies, the specific content of the activities may have influenced the observed outcomes. Therefore, it cannot be excluded that part of the effects is attributable not only to the teaching approach itself, but also to the nature of the proposed tasks.

In addition, all participants were recruited from the same school, which may limit the generalizability of the findings and represents a potential limitation of the study.

Moreover, the limited duration of each curricular session (approximately 21 min), required by the school organization, did not allow for higher training loads.

Future studies should consider including measures of biological maturation to better control for this variable, as well as incorporating specific assessments of motor competence and decision-making abilities. Moreover, future research should aim to reorganize school programs to increase physical activity during physical education lessons and to monitor internal load parameters through heart rate monitors. It would also be useful to evaluate long-term outcomes to determine whether such interventions produce lasting changes in physical activity behaviours and perceptions of physical fitness.

## 5. Conclusions

This study highlighted the effectiveness of the ecological dynamics approach, implemented through small-sided games, within curricular physical education lessons compared with traditional teaching methodologies. The findings demonstrated that SSG-based activities led to greater improvements in motor skills (e.g., explosive strength, speed, and agility), higher levels of daily physical activity, and enhanced perceived physical fitness among adolescents, thus confirming the study hypothesis.

Despite some methodological limitations, the results suggest that the ecological dynamics approach represents an effective, sustainable, and easily applicable teaching strategy in school contexts. From a practical perspective, the integration of small-sided games into physical education curricula may help teachers increase students’ active participation, promote decision-making and engagement, and foster more active lifestyles during adolescence.

## Figures and Tables

**Table 1:** Characteristics of the ecological dynamics and traditional physical education approaches.

Aspect	SSG (Ecological Dynamics Approach)	CON (Traditional Approach)
Pedagogical framework	Ecological dynamics approach based on perception-action coupling, adaptability, and continuous interaction among individual, task and environmental constraints	Teacher-centred approach based on the decomposition of skills into isolated components and the reproduction of predefined movement patterns
Main instructional aim	Promote motor adaptation, decision-making and active engagement through representative and variable learning tasks	Improve technical execution and general physical fitness through structured, prescriptive and repetitive exercises
Intervention duration	12 weeks; 2 sessions per week. Approximately 20–25 min per session dedicated to small-sided games	12 weeks; 2 sessions per week. Approximately 30 min per session dedicated to analytical drills and structured exercises
Lesson structure	Warm-up, central phase, and cool-down	Warm-up, central phase, cool-down, and stretching
Central phase content	Small-sided games (e.g., 2 vs. 2, 3 vs. 3, 4 vs. 4) derived from invasion sports (e.g., football, basketball, handball), designed to encourage continuous adaptation and decision-making	Analytical drills and structured exercises (e.g., sport-specific drills such as passing, shooting, and coordination tasks), including individual and pair-based activities aimed at technical execution and general conditioning
Task characteristics	Variable and representative tasks with ongoing manipulation of constraints (e.g., space, number of players, rules) to promote adaptive motor responses	Stable, structured, and repetitive tasks with predefined movement solutions and limited variability
Teacher role	Design representative tasks, manipulate task constraints, and provide guided feedback to support exploration and decision-making	Provide direct instruction, demonstrate exercises, and correct technical execution
Student role	Actively engage in problem-solving, explore movement solutions, and adapt behavior to changing task demands	Execute prescribed tasks and reproduce movement patterns according to teacher instructions
Feedback	Primarily guided and task-oriented feedback aimed at facilitating adaptation and decision-making	Primarily corrective feedback focused on technical accuracy
Instructional emphasis	Adaptability, engagement, and decision-making in dynamic contexts	Technical accuracy, task repetition, and physical conditioning in controlled contexts

**Table 2:** Within-Group Changes in Physical Performance Measures in Control and SSG Groups.

	CON (*n* = 22)					SSG (*n* = 26)				
Variables	PRE	POST	Δ%	ES	*p*	PRE	POST	Δ%	ES	*p*
SBJ (cm)	132.20 ± 9.88	139.31 ± 9.49	+5.38%	0.86	0.019	130.32 ± 9.76	165.53 ± 4.77	+27.04%	4.06	<0.001
5 SBJ (cm)	550.80 ± 28.72	562.31 ± 27.02	+2.09%	0.34	1.000	581.26 ± 48.35	721.05 ± 43.17	+24.01%	3.85	<0.001
20 m (s)	3.83 ± 0.14	3.73 ± 0.10	+2.61%	0.84	0.003	3.80 ± 0.12	3.53 ± 0.11	+7.11%	2.28	<0.001
10 × 5 (s)	24.03 ± 0.92	23.66 ± 0.78	+1.54%	0.48	0.021	23.90 ± 0.73	22.29 ± 0.70	+6.72%	2.21	<0.001
5-0-5 (s)	3.62 ± 0.17	3.55 ± 0.12	+1.93%	0.58	0.341	3.62 ± 0.15	3.40 ± 0.10	+6.08%	1.61	<0.001
Sit & Reach (cm)	–3.59 ± 1.03	–3.39 ± 0.86	+5.57%	0.24	1.000	–3.63 ± 0.99	–2.26 ± 0.92	+37.74%	1.42	<0.001

Note. Data presented as mean ± SD. Δ%—pre-post percentage change. ES—Cohen’s d effect size for within-group comparisons. CON—control group. SSG—Small-Sided Games group.

**Table 3:** Within-Group changes for Daily Physical Activity Levels (IPAQ-SF) in Control and SSG Group.

	CON (*n* = 22)					SSG (*n* = 26)				
Variables	PRE	POST	Δ%	ES	*p*	PRE	POST	Δ%	ES	*p*
Vigorous activity (min)	28.31 ± 6.32	30.63 ± 6.29	+8.19%	0.38	1.000	28.58 ± 7.66	41.84 ± 2.99	+46.46%	2.19	<0.001
Moderate activity (min)	17.44 ± 4.82	18.81 ± 5.43	+7.86%	0.24	1.000	16.84 ± 5.21	28.95 ± 6.36	+71.87%	2.19	<0.001
Walking (min)	47.94 ± 7.96	49.63 ± 8.21	+3.52%	0.20	0.024	49.47 ± 9.57	50.26 ± 9.43	+1.60%	0.08	0.619
Sitting (min)	529.50 ± 29.68	518.75 ± 38.23	+2.03%	0.33	0.089	526.74 ± 26.58	505.32 ± 33.23	+4.07%	0.66	<0.001

Note. Data presented as mean ± SD. Δ% indicates pre-post percentage change. ES = Cohen’s d effect size for within-group comparisons. CON = control group. SSG = Small-Sided Games group.

**Table 4:** Within-Group changes for Visual Analogue Fitness Perception Scale for Adolescents (FPVASA) in Control and SSG Groups.

	CON (*n* = 22)					SSG (*n* = 26)				
Variables	PRE	POST	Δ%	ES	*p*	PRE	POST	Δ%	ES	*p*
General fitness (level)	5.44 ± 0.51	5.50 ± 0.52	+1.10%	0.11	1.000	5.42 ± 0.51	6.47 ± 0.70	+19.37%	1.85	<0.001
Cardiorespiratory fitness (level)	5.19 ± 0.75	5.44 ± 0.73	+4.82%	0.35	1.000	5.16 ± 0.76	6.26 ± 0.56	+21.32%	1.57	<0.001
Muscular strength (level)	5.31 ± 0.48	5.50 ± 0.52	+3.58%	0.37	0.546	5.16 ± 0.50	6.42 ± 0.51	+24.42%	2.51	<0.001
Speed-agility (level)	5.25 ± 0.68	5.31 ± 0.70	+1.14%	0.09	1.000	5.16 ± 0.83	6.32 ± 0.48	+22.48%	1.68	<0.001
Flexibility (level)	5.19 ± 0.98	5.50 ± 0.73	+5.97%	0.36	0.253	4.89 ± 0.99	5.58 ± 0.69	+14.11%	0.79	<0.001

Note. Data presented as mean ± SD. Δ% indicates pre-post percentage change. ES = Cohen’s d effect size for within-group comparisons. CON = control group. SSG = Small-Sided Games group.

## Data Availability

The data presented in this study are available on reasonable request from the corresponding author due to ethical and privacy restrictions involving minors.

## Abbreviations

The following abbreviations are used in this manuscript:

MVPA	Moderate to Vigorous Physical Activity
NCDs	Chronic non-communicable diseases
IPAQ-SF	International Physical Activity Questionnaire—Short Form
FPVASA	Fitness Perception Visual Analogue Scale for Adolescents
CON	Control Group
SSG group	Experimental Group
SSG	Small-Sided Games
SBJ	Standing Broad Jump
5 SBJ	5 Standing Broad Jump
20 m	20 m sprint test
10 × 5 m	10 × 5 m Eurofit shuttle run test
COD	5-0-5 Change of Direction test
